# Eye Disease in Patients with Rheumatic Diseases: A Retrospective Observational Cohort Study

**DOI:** 10.3390/jcm12247510

**Published:** 2023-12-05

**Authors:** Ralf Altenberger, Teresa Rauchegger, Gertrud Haas, Barbara Teuchner, Michael Schirmer

**Affiliations:** 1 Clinic II, Department of Internal Medicine, Medical University of Innsbruck, 6020 Innsbruck, Austria; ralf.altenberger@gmail.com; 2 Department of Ophthalmology and Optometry, Medical University of Innsbruck, 6020 Innsbruck, Austria; teresa.rauchegger@i-med.ac.at (T.R.); gertrud.haas@tirol-kliniken.at (G.H.)

**Keywords:** ophthalmology, rheumatology, HLA-B27, immune-mediated disease, uveitis, healthcare, multidisciplinary, spondylitis, arthritis

## Abstract

Data on eye diseases in rheumatic patients are limited. The aim of this study was to retrospectively assess the prevalence of ophthalmologic diseases in patients at a rheumatology outpatient clinic who also visited the ophthalmologic clinic. For this retrospective observational cohort study, a chart review was performed according to the STROBE guidelines. In this cohort, an ophthalmologic diagnosis was made in 26.9% of the 1529 rheumatic outpatients, whereas from a rheumatologic perspective, inflammatory non-infectious diagnoses dominated, at 71.7%. From an ophthalmologic perspective, diagnoses without inflammatory pathophysiologic backgrounds dominated, at 54.9%. Inflammatory non-infectious ophthalmologic disease was diagnosed in 24.2% of patients with rheumatoid arthritis and 29.3% of patients with peripheral spondyloarthritis. Not a single rheumatoid arthritis patient was diagnosed with anterior uveitis; however, 16.5% of spondyloarthritis patients were diagnosed with anterior uveitis (*p* < 0.001). The prevalence of uveitis was 16.3% in axial and 20.1% in peripheral spondyloarthritis. In conclusion, an interdisciplinary rheumatologic–ophthalmologic setting appears justified to further improve the management of patients with rheumatic diseases.

## 1. Introduction

Spondyloarthritis (SpA) and Behçet’s disease are typical diseases requiring both rheumatologists and ophthalmologists. SpA comprises a group of pathophysiologically related diseases with different axial and peripheral manifestations. Uveitis is a recognized extraskeletal manifestation of SpA [1]. Other manifestations include psoriatic disease of the skin and ulcerative colitis or Crohn’s disease of the colon. HLA-B27 may be a risk factor for SpA [2]. Behçet’s disease is a multisystemic disease classified as variable vessel vasculitis, which involves all types of vessels, including small, medium, and large-sized veins and arteries. Typical clinical features include mucocutaneous manifestations like oral and genital aphthosis, as well as ocular manifestations like uveitis [3]. However, there are at least 10 other inflammatory rheumatic diseases that are potentially associated with uveitis [4]. Additionally, there are many more ocular manifestations that possibly present with a rheumatic disease, from keratoconjunctivitis sicca (KCS) in Sjögren’s syndrome to visual disturbances and even visual loss in giant cell arteritis (GCA). Thus, eye involvement in rheumatic diseases may have a dramatic impact on the prognosis and quality of life of patients with rheumatic diseases, whereas other eye diseases may occur independently of rheumatic disease. Therefore, all efforts to support the interactions between these two disciplines are needed, not only to find the correct diagnosis but also for treatment decisions [5]. A multidisciplinary approach was shown to improve the survival of patients with chronic diseases [6]. Data on the need for such interdisciplinary healthcare may provide an additional stimulus to support such co-operation, but larger epidemiologic studies are still missing.

The prevalences of both rheumatic and ophthalmologic diseases strongly vary depending on the populations and methods applied. Thus, a crucial challenge of epidemiologic studies is estimating the need for interdisciplinary healthcare due to the high number of different diagnoses, including the many rare diseases in both rheumatology and ophthalmology. Therefore, including both rheumatologic and ophthalmologic diseases in such an epidemiologic study to assess the need for interdisciplinary healthcare based on underlying pathophysiological principles appears to be justified.

The current literature contains little data on the prevalence of eye manifestations in specific rheumatic diseases (summarized in Table 1). In 2001, a study of 300 consecutive rheumatology patients in a large Veterans Administration Healthcare System showed that only 4% of the patients were referred due to eye manifestations, mostly anterior uveitis and KCS, in suspected rheumatic diseases [7].

The aims of this study are (1) to provide data on retrospectively assessed prevalence of ophthalmological diseases in real-world data from a rheumatology outpatient clinic, and (2) to consider the role of a multi-disciplinary rheumatology–ophthalmology clinic.

## 2. Materials and Methods

### 2.1. Study Design

The study design is a retrospective cohort analysis in the setting of a secondary/tertiary referral center at a university hospital. After written informed consent was obtained, all consecutive adult patients attending the rheumatic outpatient clinic were recruited into the cohort. Reports of ophthalmologic visits by these patients were retrospectively searched for in the hospital information system.

Retrospective data analysis was performed according to the STROBE recommendations for cohort studies [15].

### 2.2. Data Collection

Data were collected from all patients recruited between 24 September 2017 and 28 August 2020 at the rheumatology outpatient clinic who had visited the ophthalmologic clinic at any time based on documentation in the hospital information system. All data were manually excerpted from physicians’ reports until June 2022. Data included age, sex, diagnoses, and HLA-B27 status obtained from the patients’ charts, both from the rheumatology and the ophthalmology clinics.

Both rheumatic and ophthalmologic diagnoses of the patients were assigned into diagnostic groups of inflammatory non-infectious or infections and non-inflammatory diseases without malignancy or malignancies as outlined in Table 2. This method was used previously for rheumatic diagnoses [16] but was extended to ophthalmologic diagnoses in this study. In the case of multiple diagnoses, inflammatory infectious and non-infectious, as well as malignant diagnoses, were prioritized. If the assignment of a diagnosis to the underlying pathophysiological concept was unclear, a consensus was reached based on all available information from the clinical and laboratory data.

This process allowed us to assign even rare diagnoses based on a pathophysiologic concept, with the option for more detailed sequential analyses with higher patient numbers in the future. Combining rheumatic and ophthalmologic patients allowed better estimation of overlaps between subgroups.

From the rheumatic perspective, diagnoses with time of first symptom and first diagnosis were assessed together with clinical and laboratory parameters. Disease activity was assessed based on the underlying diagnosis, e.g., using the Clinical Disease Activity Index (CDAI score) for rheumatoid arthritis (RA), the Bath Ankylosing Spondylitis Diseases Activity Index (BASDAI score) for SpA, and the clinical Disease Activity in Psoriatic Arthritis Score (cDAPSA score) for Psoriatic Arthritis. Patients with arthritis, enthesitis, or dactylitis are listed as peripheral SpA, independent of the extraarticular manifestation.

Accordingly, from the ophthalmologic perspective, times of first symptom, first diagnosis, and specific laboratory data were also interrogated. If an infectious origin was identified, keratitis, scleritis, and uveitis were grouped as infectious eye diseases. Ophthalmologic diseases with an allergic pathophysiological background were listed as a separate subgroup.

### 2.3. Ethical and Privacy Considerations

Ethical approval was obtained from the ethical committee of the Medical University of Innsbruck on 15 September 2017 (AN 2017-0041 317/4.18). Patients were included in the study only after informed and written consent was obtained.

Data were stored on the University computer and password protected. To ensure the security of personal data, patients’ data were pseudonymized before statistical analyses and anonymized before publication.

### 2.4. Statistical Considerations

Data were analyzed using SPSS Statistics 25 (IBM Corporation, Armonk, NY, USA). Data were analyzed from the rheumatology and ophthalmology perspectives as indicated. Diagnostic subgroups were analyzed separately if they were important for later discussion.

For the patients’ characteristics, categorical data are shown with counts and percentages. Age, as a metric variable, was tested for normal distribution using the Kolmogorov–Smirnov test. As both variables did not follow a normal distribution, the median and interquartile range were used for descriptive purposes.

To analyze possible differences between subgroups, the chi-squared test or Fisher’s exact test (if a cell count was below 5) was used as described. The Mann–Whitney U test was used to compare median ages between groups. *p*-values < 0.05 were considered significant. Bonferroni correction was applied where indicated.

## 3. Results

**Figure 1:**
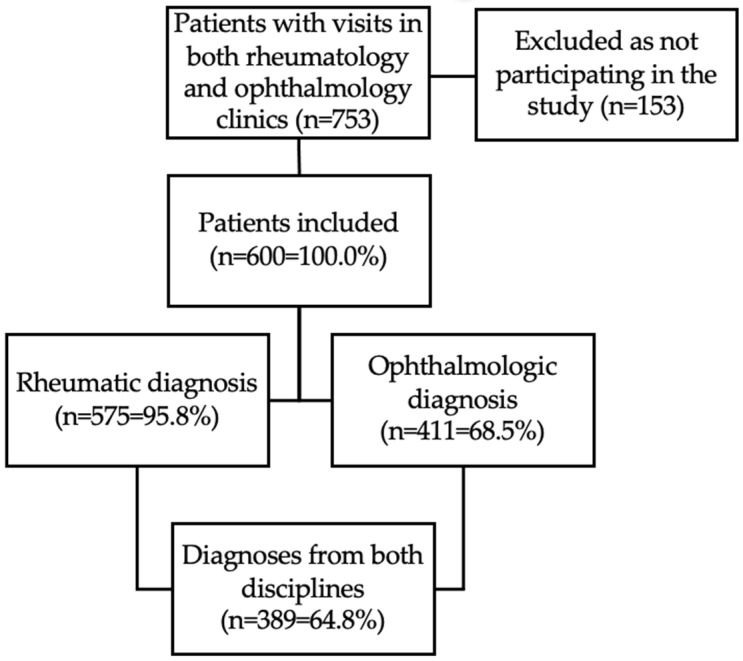
Flow chart showing the selection of charts with the overlap of rheumatic and ophthalmologic diagnoses.

As depicted in Figure 1, 753 of 1529 patients at the rheumatic outpatient clinic were identified as visiting the ophthalmologic clinic. After manual verification, 600 patients participated in the ethically approved study.

Out of the 600 patients, 575 patients (95.8%) had a rheumatic diagnosis and 411 patients (68.5%) had an ophthalmologic diagnosis. The remaining 4.2% and 31.5% of the patients had no rheumatic or ophthalmologic diagnoses, respectively. Out of the 1529 patients, 411 patients (26.9%) had an ophthalmologic diagnosis, while 389 patients (64.8% of all patients included) had both a rheumatic and an ophthalmologic diagnosis.

### 3.1. Patient and Disease Characteristics

#### 3.1.1. The Rheumatologist’s Perspective

The characteristics of patients with a rheumatic diagnosis and a history of an ophthalmologic visit are shown in Table 3. Within this cohort of patients, inflammatory non-infectious rheumatic disease was more frequent than a non-inflammatory diagnosis without malignancy (71.7% vs. 27.5%, *p* < 0.001).

Most patients with rheumatic disease were diagnosed with rheumatoid arthritis (RA), followed by peripheral SpA, and axial SpA (with 16.5, 14.3, and 12.0% of all patients with rheumatic diagnoses, respectively). RA was more frequent than axial SpA (*p* = 0.025) but was not significant when compared to pSpA (*p* = 0.288). Women dominated in all diagnostic groups, except gout (with 17.2%) and Behçet’s disease (with 46.7%). At 217 months (18.1 years), patients with SLE had the longest median time since diagnosis.

The disease activities of patients with an inflammatory non-infectious disease at the last available visit are described in Supplementary Table S1. Gout had the highest percentage of patients with an active disease, followed by Sjögren’s syndrome. PMR/GCA had the lowest percentage of patients with active disease, while RA had the second lowest. Axial SpA had a higher rate of active disease compared to RA (*p* < 0.001) and PMR/GCA (*p* = 0.002). On the other hand, PMR/GCA had the highest remission rate, followed by Behcet’s disease and RA. Sjögren’s disease had the lowest remission rate, while axial SpA had the second lowest. The remission rate for axial SpA was lower than that for RA (*p* = 0.001).

#### 3.1.2. The Ophthalmologist’s Perspective

The characteristics of patients with ophthalmologic diseases are shown in Table 4. Patients with an inflammatory non-infectious disease were fewer than those with a non-inflammatory diagnosis without malignancy (35.0% vs. 54.7%; *p* < 0.001).

Patients with an inflammatory non-infectious eye disease had a lower median age compared with those with a non-inflammatory eye disease without malignancy (56.0 and 69.0 years, respectively; *p* < 0.001). The frequency of KCS differed in both anterior uveitis and keratitis (*p* < 0.001 each).

#### 3.1.3. Coincidence of Rheumatic and Ophthalmologic Diagnoses

Out of the 389 patients with diagnoses from both disciplines, 350 patients (90.0%) had an inflammatory non-infectious or non-inflammatory diagnosis without malignancy. There was no coincidence between the two diagnostic groups (*p* = 0.511). Detailed numbers and percentages are shown in Table 5.

### 3.2. Prevalences of Inflammatory Non-Infectious Diagnoses of the Other Respective Discipline in Specific Diagnoses

With no coincidence between rheumatic and ophthalmologic inflammatory non-infectious diagnoses, each inflammatory non-infectious diagnosis from both the rheumatic and the ophthalmologic perspectives was further analyzed (as detailed in Table 6). As expected, patients with Sjögren’s syndrome had the highest percentage of inflammatory non-infectious eye manifestations, as KCS was frequent. From the ophthalmologic perspective, keratitis, anterior uveitis, and KCS were the diagnoses with the highest percentage of underlying inflammatory non-infectious rheumatic disease.

### 3.3. Ophthalmologic Diagnoses in Rheumatoid Arthritis and Spondyloarthritis

RA and SpA were the most prevalent inflammatory non-infectious rheumatic diseases (Table 7). For the comparison of RA patients with SpA, axial SpA and pSpA were combined into the SpA group.

More RA patients had KCS (17.6%) and keratitis (8.5%) than SpA, although this difference was not significant. Of the SpA patients, 16.5% were diagnosed with anterior uveitis (*p* < 0.001), whereas not a single RA patient was diagnosed with anterior uveitis. The other anatomical uveitis types comprised a low percentage of each rheumatic disease (<3%).

### 3.4. Rheumatologic Diagnoses in Ophthalmologic Manifestations

Table 8 shows the association between ophthalmologic diagnoses and rheumatic diagnoses. For the comparison with KCS and keratitis, all subgroups of uveitis were combined into the uveitis group.

Patients with uveitis were diagnosed with RA less frequently than patients with KCS (7.0% vs. 21.3%, *p* = 0.056) and patients with keratitis (7.0% vs. 46.2%, *p* = 0.003). Peripheral SpA was diagnosed more often in patients with uveitis than in patients with KCS (32.6% vs. 11.4%, *p* = 0.008) and patients with keratitis (32.6% vs. 0.0%, *p* = 0.025).

### 3.5. Prevalence of HLA-B27 in Uveitis Subgroups

HLA-B27 test was positive exclusively in patients diagnosed with anterior uveitis, but not in patients with intermediate uveitis or posterior uveitis. Overall, the percentage of HLA-B27 positivity was higher in patients with anterior uveitis compared with those with the other anatomical types of uveitis (*p* = 0.002 compared with intermediate, *p* = 0.019 compared with posterior, and *p* = 0.029 compared with panuveitis. Following Bonferroni correction, only comparisons with intermediate uveitis showed significant differences). A total of 304 rheumatic patients with eye diseases (50.7%) were tested for HLA-B27 status. Out of these, HLA-B27 status was available for 48 patients with uveitis but was not available for 7 patients with uveitis (as detailed in Table 9).

## 4. Discussion

As many as 26.9% of all consecutive rheumatic patients were diagnosed with an ophthalmologic manifestation or disease at the ophthalmologic clinic. This percentage is in line with the available literature on patients with specific rheumatic diagnoses like RA and SpA (as shown in Table 1) but may be even higher, as this study did not assess ophthalmologic visits occurring outside of the hospital. This high number of ophthalmologic diagnoses in rheumatic patients may argue for a multi-disciplinary rheumatology–ophthalmology clinic. The lower percentage of 4% ophthalmologic diagnoses in a smaller rheumatic cohort may be explained by the cohort’s characteristics [7]. The consecutive patients included in this cohort can be considered typical and representative of a secondary and tertiary rheumatology center with co-operating ophthalmologic and rheumatologic services.

To the best of our knowledge, the prevalence of ophthalmologic diseases in a large rheumatologic cohort has not been determined previously. From the rheumatologic perspective, inflammatory non-infectious diagnoses were more frequent than non-inflammatory diagnoses without malignancy (71.7% vs. 27.5%, *p* < 0.001; Table 3), whereas the majority of ophthalmologic diagnoses were more often non-inflammatory without malignancy compared with inflammatory non-infectious diseases (54.7% vs. 35.0%, respectively, *p* < 0.001; Table 4). We then wondered whether a non-inflammatory rheumatic diagnosis would exclude an inflammatory non-infectious ophthalmologic diagnosis. This thought can be negated, as 41.8% of patients with a non-inflammatory rheumatic diagnosis had an inflammatory non-infectious ophthalmologic diagnosis (Table 5). Certainly, non-inflammatory ophthalmologic diagnoses without malignancy may be underestimated, as they are also managed in medical services other than the university hospital. In these patients, pure ophthalmologic counseling with the inclusion of recommendations for self-management can lead to better-informed patients.

From the rheumatology perspective, inflammatory non-infectious ophthalmologic diagnoses may be important when deciding on the immunosuppressive management of rheumatic disease. Regarding specific diagnoses, anterior uveitis was frequent in SpA, at 16.5%, but was not diagnosed in RA (*p* < 0.001), whereas the other anatomical sites of uveitis (intermediate, posterior, and panuveitis) were equally distributed between RA and SpA (Table 7). This confirms the possible role of anterior uveitis in axial SpA, as already mentioned in the existing literature [17], whereas RA does not seem to tend to develop anterior uveitis. At 16.3% and 20.1%, the prevalences of any anatomical site of uveitis are lower in both axial and peripheral SpA patients, respectively, in this cohort compared with those described in a literature review from 2006, at 32.2% and 33.2% (Table 1) [13]. This reduced prevalence of uveitis in our SpA patients may be a consequence of the improved management options for SpA over the past 15 years, especially with the use of biological disease-modifying antirheumatic agents. Still, this fact emphasizes the need for rheumatologists to consider ocular manifestations. We expected a higher percentage of KCS, which is the most common ocular manifestation of RA, in RA patients (Table 1). However, KCS is not always triggered by an autoimmune process, and many different factors can lead to KCS [18], which also means that a KCS diagnosis in RA patients does not necessarily indicate secondary Sjögren’s syndrome. Thus, KCS does not always retrospectively refer to the inflammatory non-infectious or non-inflammatory pathophysiologic group without malignancy, which can only be solved by the ophthalmologists in a prospective study design. In this cohort, episcleritis (n = 2) and scleritis (n = 1) were rare. Considering the given prevalences (Table 1), we expected to find a higher number of patients with episcleritis.

In clinical practice, rheumatic and ophthalmologic manifestations do not always occur simultaneously. In fact, an ophthalmologic manifestation may occur before, together with, or after the onset of a rheumatic disease. Because of this, considering ocular manifestations as early as possible and consulting an ophthalmologist before visual impairment or even blindness occurs is one of the crucial challenges for rheumatologists. If suspected, even for patients with non-inflammatory rheumatic diagnoses, an ophthalmologic visit might help to detect an inflammatory ocular disease earlier. Vice versa, 73.5% of patients with an inflammatory non-infectious eye disease have a concomitant inflammatory non-infectious rheumatic disease. Additionally, rheumatologists can provide the infrastructure for education and application of intravenous and subcutaneous drugs. As about 40% of patients with an inflammatory rheumatic diagnosis also have an inflammatory ocular diagnosis, these patients usually need specific treatment decisions (especially concerning the strength of their immunosuppression). Therefore, both disciplines may see a single patient to manage different manifestations of the same disease, two separate disease entities, or even to manage ocular side-effects of medications for rheumatic diseases. Nevertheless, it may be helpful for both specialists to have their patients rapidly assessed by the co-operating specialist. This is made possible by the commitment of both the rheumatologists and the ophthalmologists, and not only provides an advantage for earlier diagnosis, as seen in the fast-track referral for GCA [19], but also for monitoring purposes during the disease courses. In doing so, relapses may be detected earlier.

The most important limitation is probably the retrospective design of this analysis, with manual chart review of unstructured clinical data, which may lead to incomplete or even missing datasets. For example, the HLA-B27 status was not available for all patients with uveitis. Additionally, data from ophthalmologic examinations performed outside of the hospital were not accessible. As a consequence, the subgrouping of patients according to their underlying pathophysiological mechanisms of rheumatic and ophthalmologic manifestations is sometimes difficult. Both inflammatory and non-inflammatory diagnoses were therefore grouped as “inflammatory”. This leads to a certain bias, but it can be argued that the higher clinical relevance of inflammatory compared to non-inflammatory diseases justifies this grouping. For example, a patient with SLE and osteoarthritis is listed as “inflammatory”. Another weakness of the study is certainly its relatively small number of 600 patients, partly including rare diseases. Therefore, subgrouping can be helpful to allow comparisons between the inflammatory non-infectious and non-inflammatory without malignancy subgroups. This will be less important if extensive data from numerous tertiary referral centers across various countries all over the world can be pooled, as done by the “AIDA Network uveitis and scleritis registries” [20]. This study was limited by M.S. being the only recruiting investigator. Statistical analyses with adjustments for age and gender were therefore not performed.

## 5. Conclusions

Ocular diseases were observed in more than every fourth adult rheumatic patient, particularly, but not exclusively, those with inflammatory non-infectious rheumatic diseases. Multidisciplinary clinics with rheumatologists and ophthalmologists may improve outcomes; however, further research is required.

## Figures and Tables

**Table 1:** The prevalences of ophthalmologic diseases in the normal population compared with their prevalences in selected rheumatic diseases (references in parentheses).

Ophthalmologic	Prevalence		
Disease	In General Population (%)	In Rheumatic Disease (%)	
KCS	5–50 [8]	RA	18–90 [9]
Episcleritis	0.041 [10]	RA	0.17–3.7 [11]
Scleritis	0.0051 [10]	RA	0.2–6.3 [12]
Uveitis	0.07–0.7 [13]	Axial SpA	32.2 [14]
		Periperal SpA	33.2 [14]
		Psoriatic arthritis	25.1 [14]
		Reactive arthritis	25.6 [14]

KCS, keratoconjunctivitis sicca; RA, rheumatoid arthritis; SpA, spondyloarthritis.

**Table 2:** Examples of rheumatic and ophthalmologic diagnoses subgroups based on the pathophysiological concepts of inflammation, infection, and malignancy.

Pathophysiology	Rheumatology	Ophthalmology
Inflammatory, non-infectious	RA, SpA, PMR/GCA, SLE, Sjögren’s syndrome, gout, pseudogout	KCS, keratitis (e.g., peripheral ulcerative keratitis, Mooren’s ulcer, keratitis marginalis), uveitis, episcleritis, scleritis
Infections	Primary infection ^1^, secondary to infection, diffuse peri-infectious MSK symptoms	infectious keratitis, uveitis or scleritis, blepharitis, hordeolum, conjunctivitis
Non-inflammatory, without malignancy	Osteoarthritis, osteoporosis, fibromyalgia	Primary glaucoma, cataract, macular degeneration, vitreous detachment, retinal detachment, ptosis, subconjunctival hemorrhage, dermatochalasis
Malignancies	Primary tumors ^2^, metastases, diffuse paraneoplastic MSK symptoms	Basalioma, melanoma

^1^ e.g., viral, bacterial, or fungal infections. ^2^ e.g., bone tumors. GCA, giant cell arteritis; KCS, keratoconjunctivitis sicca; MSK, musculoskeletal; PMR, polymyalgia rheumatica; RA, rheumatoid arthritis; SpA, spondyloarthritis; SLE, systemic lupus erythematosus.

**Table 3:** Patient characteristics based on etiologic subgroups and rheumatic diagnoses (with diagnoses in alphabetic order; percentages refer to the 575 patients with rheumatic diagnosis). Age and disease duration are shown as median and interquartile range.

	N (%)	Age (years)	Gender (% ♀)	Time since Diagnosis (months)
Inflammatory				
• Non-infectious	412 (71.7)	60.5 (51.0–73.0)	62.4	47.0 (22.0–137.8)
- Axial SpA	69 (12.0)	53.0 (41.5–61.0)	62.3	43.0 (23.5–184.5)
- Behçet’s	15 (2.6)	49.0 (39.0–55.0)	46.7	55.0 (20.0–235.0)
- Gout	29 (5.0)	64.0 (51.5–79.5)	17.2	24.0 (21.5–40.5)
- pSpA	82 (14.3)	55.5 (50.0–66.0)	59.8	24.0 (15.0–54.0)
- RA	95 (16.5)	72.0 (59.0–79.0)	70.5	120.0 (48.0–120.0)
- PMR/GCA	48 (8.3)	76.0 (68.0–80.0)	60.4	32.0 (23.0–60.0)
- Sjögren’s	10 (1.7)	66.0 (50.5–74.8)	80.0	48.5 (9.0–206.0)
- SLE	8 (1.4)	49.0 (45.3–56.5)	87.5	217.0 (147.5–319.0)
- Others *	56 (9.7)	53.0 (40.0–62.0)	69.1	22.0 (13.0–33.0)
• Infections	4 (0.7)	61.0 (57.8–65.8)	75.0	18.0 (11.5–24.5)
Non-inflammatory				
• Without malignancy	158 (27.5)	60.0 (46.8–71.0)	70.3	19.0 (12.0–30.0)
Malignancies	1 (0.2)	75.0	100.0	24.0
Total	575	60.0 (50.0–73.0)	64.7	32.0 (18.0–86.0)

* others include juvenile idiopathic arthritis, granulomatosis with polyangiitis, idiopathic vasculitis, large vessel vasculitis, CREST syndrome, SAPHO syndrome, RS3PE syndrome, juvenile dermatomyositis, retroperitoneal fibrosis, sarcoidosis, systemic sclerosis, SHARP syndrome, chondrocalcinosis, tendinitis, tendovaginitis, enthesitis. GCA, giant cell arteritis; PMR, polymyalgia rheumatica; RA, rheumatoid arthritis; SLE, systemic lupus erythematosus; SpA, spondyloarthritis.

**Table 4:** Patient characteristics based on diagnostic subgroups (inflammatory, non-inflammatory) and ophthalmologic diagnoses (in alphabetical order, percentages refer to total number of patients with ophthalmologic diagnosis). Patients with keratitis and KCS are listed as keratitis, diseases diagnosed in less than 1% of the patients were summarized as others. Age is given with median and interquartile range.

	N (%)	Age (years)	Gender (% female)
Inflammatory			
• Non-infectious	144 (35.0)	56.0 (44.0–68.0)	63.9
- Anterior uveitis	33 (8.0)	53.0 (35.5–60.0)	39.1
- Intermediate uveitis	12 (2.9)	40.5 (28.8–71.5)	58.3
- KCS	64 (15.6)	57.5 (47.3–71.8)	76.6
- Keratitis	13 (3.2)	63.0 (56.0–79.5)	100
- Panuveitis	5 (1.2)	58.0 (40.5–62.0)	40.0
- Posterior uveitis	5 (1.2)	58.0 (39.5–71.5)	100
- Others *	12 (2.9)	49.0 (43.5–56.5)	33.3
• Infections	38 (9.2)	55.0 (45.8–62.0)	55.3
Non-inflammatory			
• Without malignancy	225 (54.7)	69.0 (53.0–77.0)	67.1
Malignancies	1 (0.2)	67.0	100
Allergic	3 (0.7)	40.0 (38.5–45.0)	100
Total	411	61.0 (49.0–74.0)	65.2

* others include arteritic anterior ischemic optic neuropathy, conjunctivitis, episcleritis, neuroretinitis (non-Behcet’s disease-associated), retinal vasculitis, and scleritis. KCS, keratoconjunctivitis sicca.

**Table 5:** Two-way table of patients with rheumatic (lines) and ophthalmologic (columns) diagnoses, with percentages of all patients with rheumatic diagnoses shown in the respective line before the slash and the percentages of all patients with ophthalmologic diagnoses of the respective column after the slash. N; number.

	Ophthalmologic Diagnosis	Inflammatory Non-Infectious	Non-Inflammatory without Malignancy
Rheumatic Diagnosis			
Inflammatory non-infectious (%/%)		N = 100 (37.9/73.5)	N = 164 (62.1/76.6)
Non-inflammatory without malignancy (%/%)		N = 36 (41.8/26.5)	N = 50 (58.2/23.4)

**Table 6:** Inflammatory non-infectious diagnoses from (A) the rheumatic and (B) the ophthalmologic perspectives, with percentages of inflammatory non-infectious diagnoses diagnosed by the other discipline (calculated from the total number of patients with the specific diagnosis).

(A) Rheumatic Diagnoses	% with Inflammatory Non-Infectious Eye Disease
Sjögren‘s syndrome	50.0
Behçet’s disease	33.3
pSpA	29.3
RA	24.2
Axial SpA	23.2
PMR/GCA	10.6
Gout	10.3
SLE	0.0
**(B)** **Ophthalmologic Diagnoses**	**% with Inflammatory Non-Infectious Rheumatic Disease**
Keratitis	76.9
Anterior uveitis	69.7
KCS	67.2
Posterior uveitis	60.0
Intermediate uveitis	41.7
Panuveitis	40.0

**Table 7:** Comparison between RA, axial SpA, pSpA, and SpA in specific ophthalmologic eye diseases. Percentages are given for all patients with a specific rheumatic diagnosis who also obtained an ophthalmologic diagnosis (patients without an ophthalmologic diagnosis were excluded).

	RA (n = 71)	Axial SpA (n = 43)	pSpA (n = 60)	SpA (n = 103)
Inflammatory				
• Non-infectious	32.4	44.2	38.3	40.8
- Anterior uveitis	0	16.3	16.7	16.5
- Intermediate uveitis	2.8	0	1.7	1.0
- KCS	17.6	14.0	11.6	12.6
- Keratitis	8.5	7.0	0	2.9
- Panuveitis	1.4	0	0	0
- Posterior uveitis	0	0	1.7	1.0
- Others	1.4	7.0	6.7	6.8
Infections	4.2	7.0	10.0	8.7
Non-inflammatory				
• Without malignancy	63.4	48.8	51.6	50.5
Malignancies	0	0	0	0

**Table 8:** Comparison of KCS, keratitis, and uveitis in the rheumatic diagnoses. Percentages are given for all patients with specific ophthalmologic diagnoses who also obtained a rheumatic diagnosis (patients without a rheumatic diagnosis were excluded).

	KCS (%) (n = 61)	Keratitis (%) (n = 13)	Uveitis (%) (n = 43)
Inflammatory			
• Non-infectious	70.5	76.9	76.7
- Axial SpA	9.8	23.1	16.3
- Behcet’s	3.3	0	7.0
- Gout	1.6	0	2.3
- Peripheral SpA	11.4	0	32.6
- PMR/GCA	4.9	0	2.3
- RA	21.3	46.2	7.0
- Sjögren’s/SLE	8.2	0	0
- Others	9.8	7.7	9.3
Infections	0	0	0
Non-inflammatory			
• Without malignancy	29.5	23.1	23.3
Malignancies	0	0	0

**Table 9:** HLA-B27 positivity in patients in the four anatomic uveitis subgroups, with the number of HLA-B27 positives before and the total numbers after the slash (with percentage positivity given in parentheses).

	HLA-B27 +	Undetermined
Anterior uveitis	18/30 (60.0%)	3
Intermediate uveitis	0/9 (0.0%)	3
Posterior uveitis	0/5 (0.0%)	0
Panuveitis	1/4 (25.0%)	1
Uveitis, total	19/48 (39.6%)	7

## Data Availability

The anonymized data are available from the authors on request.

## Supplementary Materials

The following supporting information can be downloaded at: https://www.mdpi.com/article/10.3390/jcm12247510/s1, Table S1. Disease activity of patients with inflammatory non-infectious diseases at last visit (in alphabetical order, percentages calculated based on all patients with the specific disease).
