# Supplementary material for: Eye Disease in Patients with Rheumatic Diseases: A Retrospective Observational Cohort Study
Source: J Clin Med. 2023 Dec 5;12(24):7510. doi: 10.3390/jcm12247510 (PMC10744173; doi:10.3390/jcm12247510)
Supplement: Supplementary file 1 [file jcm-12-07510-s001.zip › jcm-2675697-supplementary.pdf]

Table S1 Disease activity of patients with inflammatory non-infectious diseases at last visit (in alphabetical order, percentages calculated out of all patients with the specific disease)

	**Active disease (%)**	**Low disease activity (%)**	**Full remission (%)**	**Not categorized (missing data) (%)**
Axial SpA	33.3	34.8	27.6	4.3
Behçet's disease	26.7	20.0	53.3	0
Gout	44.8	20.7	34.5	0
Peripheral SpA	13.4	38.8	44.8	3.0
PMR/GCA	8.3	12.5	79.2	0
RA	10.6	36.8	52.6	0
Sjögren's/SLE	38,9	44.4	16,7	0
others	33.8	20.6	23.5	22.1
